# Wealth inequality in the prehispanic northern US Southwest: from Malthus to Tyche

**DOI:** 10.1098/rstb.2022.0298

**Published:** 2023-08-14

**Authors:** Timothy A. Kohler, Darcy Bird, R. Kyle Bocinsky, Kelsey Reese, Andrew D. Gillreath-Brown

**Affiliations:** ^1^ Department of Anthropology, Washington State University, Pullman, Washington 99164, USA; ^2^ Santa Fe Institute, Santa Fe, NM 87506, USA; ^3^ Crow Canyon Archaeological Center, Cortez, CO 81321, USA; ^4^ Department of Archaeology, University of Durham, Durham DH1 3LE, UK; ^5^ WA Franke College of Forestry and Conservation, University of Montana, Missoula, MT 59812, USA; ^6^ Environmental Stewardship Group, Los Alamos National Laboratory, Los Alamos, NM 87545, USA

**Keywords:** Neolithic, wealth inequality, US Southwest, archaeology, demography, palaeoclimates

## Abstract

Persistent differences in wealth and power among prehispanic Pueblo societies are visible from the late AD 800s through the late 1200s, after which large portions of the northern US Southwest were depopulated. In this paper we measure these differences in wealth using Gini coefficients based on house size, and show that high Ginis (large wealth differences) are positively related to persistence in settlements and inversely related to an annual measure of the size of the unoccupied dry-farming niche. We argue that wealth inequality in this record is due first to processes inherent in village life which have internally different distributions of the most productive maize fields, exacerbated by the dynamics of systems of balanced reciprocity; and second to decreasing ability to escape village life owing to shrinking availability of unoccupied places within the maize dry-farming niche as villages get enmeshed in regional systems of tribute or taxation. We embed this analytical reconstruction in the model of an ‘Abrupt imposition of Malthusian equilibrium in a natural-fertility, agrarian society’ proposed by Puleston *et al*. (Puleston C, Tuljapurkar S, Winterhalder B. 2014 *PLoS ONE* **9**, e87541 (doi:10.1371/journal.pone.0087541)), but show that the transition to Malthusian dynamics in this area is not abrupt but extends over centuries

This article is part of the theme issue ‘Evolutionary ecology of inequality’.

## 1. Introduction

For over two centuries we have connected the name of Reverend Thomas Malthus with the proposition that population growth tends to outstrip growth in the supply of food [1]. In the absence of ‘preventive checks’ on population increase such as later marriage or birth control, our tendency towards exponential growth, he argued, leads to the human miseries of hunger, disease and war that provide ‘positive checks’ on population.

In its simplest form, of course, this model has not withstood the test of time very well, not least because Malthus was writing at the dawn of the Industrial Revolution and the massive increase in output it generated. (Perhaps we can attribute this to Tyche, the goddess of chance.) Thus began a period in which technological progress exceeded the rate of population growth (even though that too has been very high), leading to a prosperity possibly unequalled in history [2]. Malthus himself, in his later work, brought substantial revisions to his earlier thesis [3]. The disconnect between Malthusian concerns and the experience of the Industrial Revolution notwithstanding, social scientists and perhaps especially archaeologists and demographers of ‘natural fertility’ societies continue to find inspiration in Malthus. The current renaissance of Malthus studies (e.g. [4–7]) is perhaps not surprising in a world just crossing the 8 billion threshold. In this paper we examine the extent to which a Malthus-inspired model [8] fits the known facts of the prehispanic northern Pueblo world. We will provide evidence that the period of Puebloan history most marked by high inequality is both the culmination of a period of high population growth in which large population sizes had begun to constrain mobility, inducing circumscription [9], and also the culmination of a long period of potential high agricultural surpluses made possible by favourable climates.

This is an exercise in model-based archaeology [10]. The model on which we focus is in the evolutionary–ecological tradition as defined by Winterhalder [11]. Unlike earlier ecosystem simulation models, such models focus on adaptive designs and strategies, typically operating in a theoretical/deductive mode [11]; the first such models eventually paved the way for the field of behavioural ecology. The model (to be examined through its application to sets of data drawn from Puebloan prehistory) is intentionally simple. Realism is sacrificed in service of generality, and such models cannot be expected to reproduce any particular historical sequence in any detail. The model resolutely focuses on the grand outlines of the ‘variables’ that played such a large role in processual archaeology, such as demography, subsistence, nutritional stress, and climatic variability. But, as we will see, such framing variables interact in ways that affect degree of social inequality and violence—typical concerns of a more contemporary archaeology. Indeed, the utility of models such as that we will shortly present lies in their ability to throw into relief the human, social and cultural factors that were important in forming specific real histories [12]. This is accomplished by interpreting what can metaphorically be called the residuals: what are those aspects of the focal societies that do not fit the model, and why do they not fit?

Yet at the same time the simplicity and generality of models like [8] underscore similarities in trajectory among very diverse human societies. In times of profound division by politics, education and wealth, within and between nations, it is important to remember what all people and societies have in common. By building models and critically looking for their logical consequences in the archaeological record, we engage in a double hermeneutic (borrowing the term but not its meaning from Giddens [13]) in which we seek to simultaneously improve our models and our understanding of the archaeological record.

## 2. A model for food- and space-limited agrarian population dynamics

Cedric Puleston *et al.* [8] construct a closed-form model (henceforth the PTW model) for scenarios in which farmers encounter a rich but previously unused landscape and grow to the carrying capacity of that environment more or less quickly. This carrying capacity is not directly set by the model but emerges from the interactions between the productive capacity of the environment, which is parameterized and invariable across time and space within a run, and an age-structured population. Several quantities emerge as the model is iterated: age-specific fertility and survivorship of the population; the population age structure and its total size; the age-specific labour availability and food requirements; and the important food ratio (*E*), which is the ratio of food available divided by the baseline requirements of the entire population (both customarily expressed in kilocalories). The disaggregation of population cohorts, each with its specific food requirements and labour potential, differentiates this from more traditional (and simpler) models in the tradition of Lotka & Verlhurst, as does the centrality of the food ratio, *E*, which both ‘drives vital demographic rates [and] also serves as a measure of human welfare’ [8, p. 3].

Space precludes full description of model complexities, for example the interactions between *E* and survival and fertility (though these take predictable forms); we will focus on model outputs. The standard temporal pathway produces population trajectories that Puleston *et al.* [8] divide into three phases. First comes a ‘Copial Phase’, a term coined to describe the period of relatively rapid population growth between initial settlement and the point at which the population first experiences suboptimal food intake. This is followed by a ‘Malthusian Transition Interval’ during which food limits on reproduction and survival become increasingly severe. Finally the population enters an equilibrium or ‘Malthusian Phase’ of extended misery in which growth ceases as food limits cause rates of birth and death to converge.

When realistic rates for demography and production are chosen, a key and somewhat unexpected finding of the model is the brief duration of the Malthusian Transition Interval relative to the Copial Phase. (The duration of the final Malthusian Phase is potentially infinite, since invention and innovation are not possible in this model.) Although *E* (which can also be thought of as the potential production surplus) declines gradually during the Copial Phase, its effects on life expectancy and mortality of infants and youths take hold suddenly and only as *E* falls below 1.0. In typical parameterizations the Copial Phase lasts three or four centuries whereas the Malthusian Transition Interval lasts only five or six decades.

We now consider the applicability of this model to the Pueblo societies of the upland US Southwest, drawing heavily on findings of the Village Ecodynamics Project (VEP). The VEP emphasized reconstruction of demography in its two main study areas, roughly equivalent to the central Mesa Verde (CMV) area (VEPIIN) [14,15] and the northern Rio Grande (VEPIIS) [16]. An additional focus was reconstruction of annual maize productivity in the northern area [17] and annual reconstruction of the spatial niche for dry-farmed maize for a much larger portion of the upland US Southwest (UUSS) [18,19]. The third main emphasis of the project was to use agent-based simulations [20] to model spatial behaviours as households attempt to satisfice their needs for calories, protein, wood and water on productivity landscapes that are constantly in flux owing to climatic variability and human use [21,22]. Modelling on these landscapes was also extended to the formation of groups and leadership [23] and competition over agricultural lands [24]. Among several VEP spin-offs was a series of papers examining the development of wealth inequality in various spatial and temporal subsets of the northern Southwest [25–27] and a low-frequency reconstruction of summer temperatures in the Southwest for the last 5000 years [28]. These analyses provide data and context for the arguments below.

To outline our main argument, to be documented in the following sections: for all of its endlessly fascinating counter-currents and human drama, the trajectory of Pueblo societies from *ca* 500 BC to AD 1500 was quite simple, and resonates strongly with the Malthusian story described by PTW. Innovations in food production beginning with the introduction of maize to the southern US Southwest in (likely) the fourth millennium BC [29], and its slow spread northward in conjunction with selection for production under novel photoperiods and temperature regimes [30,31] increased the regional carrying capacity and led to marked increases in crude birth rates and somewhat smaller increases in life expectancy at age 15. These coincided mostly strongly between about AD 500 and AD 1000, to produce the rapid population growth expected in a Neolithic Demographic Transition (NDT) [32,33]. Given a more or less constant supply of arable lands, this in turn led to increasing competition for superior fields and to decreases in mobility towards the end of the highest-growth portion of the NDT.

Around the same time—the mid-to-late ninth century—the Chaco regional system supplied a novel sociopolitical and ceremonial model offering an attractive pathway for continued growth and connection that overcame scale-related impediments to coordination within and between communities. This is not the place to discuss Chacoan characteristics in detail (see, e.g. [34]); suffice it to say that their largest buildings—Great Houses and Great Kivas—vastly surpassed their predecessors in size, and the spatial scale of the area joined by the regional system was correspondingly greater than any previous inter-village polity. Chaco was made possible by decreased mobility and relatively high maize production in a regional environment featuring considerable variability in maize production across space and from year to year. Its people and institutions must have tolerated, and may have celebrated, relatively high levels of social inequality.

By the mid-1100s, however, high-frequency drought in many portions of the Southwest [19,35] reduced the scale of this system, and by *ca* AD 1200 low-frequency climates began to be dominated by La Niña-like conditions that likely decreased the carrying capacity of the entire US Southwest for farmers [28]. This could not have come at a worse time for the Southwest's farming communities, who by this time were much more numerous than they had ever been. Bioarchaeologists report that childhood hypoplasia, a marker for nutritional stress and/or disease, increased by about 25% from Pueblo I times (*ca* AD 800) to Pueblo II times (*ca* AD 1100) [36]. In another study that ranked a number of Southwestern societies in terms of their pathology and mortality profiles, late (Pueblo II and III) contexts from Mesa Verde rank near the bottom, above only Arroyo Hondo in the northern Rio Grande in the fourteenth century [37]. The tempo of intra-regional movement in the northern Southwest increased in the 1200s, and by around AD 1280 the northern Pueblo area was depopulated amid widespread violence in favour of areas along the Rio Grande and a few other areas to the south. Outside of the area we focus on, populations in the Hohokam and Mimbres Mogollon areas were also greatly reduced.

If we focus on the CMV region as approximated by the VEPIIN area (see locational map in [15]), we can identify a Copial Phase from *ca* AD 600 (when this area was first occupied in force) to *ca* AD 1000, and a Malthusian Transition Interval from then to about AD 1300. Thereafter, until the arrival of the Spanish, Pueblo societies (and probably those of most of the Hohokam and Mogollon regions as well) entered a Malthusian Phase. Societies in this phase, however, were certainly not lacking in innovation—one of many ways in which this real history departs from the PTW model. In the Pueblo world the boundaries between these intervals were also not as sharp as in the model, and the tendencies they mark occurred at slightly different times in different places and take on slightly different character as well. One advantage of the model is to help us recognize similarities in the numerous complicated histories we encounter across the Southwest.

In the remainder of this article we document the assertions in the previous paragraphs and consider the reasons why the real societies we discuss were both more resilient, and more tragic, than those imagined by the PTW model.

### (a) Population size in the prehispanic Southwest trended strongly upwards until approximately AD 1200

**Figure 1.**
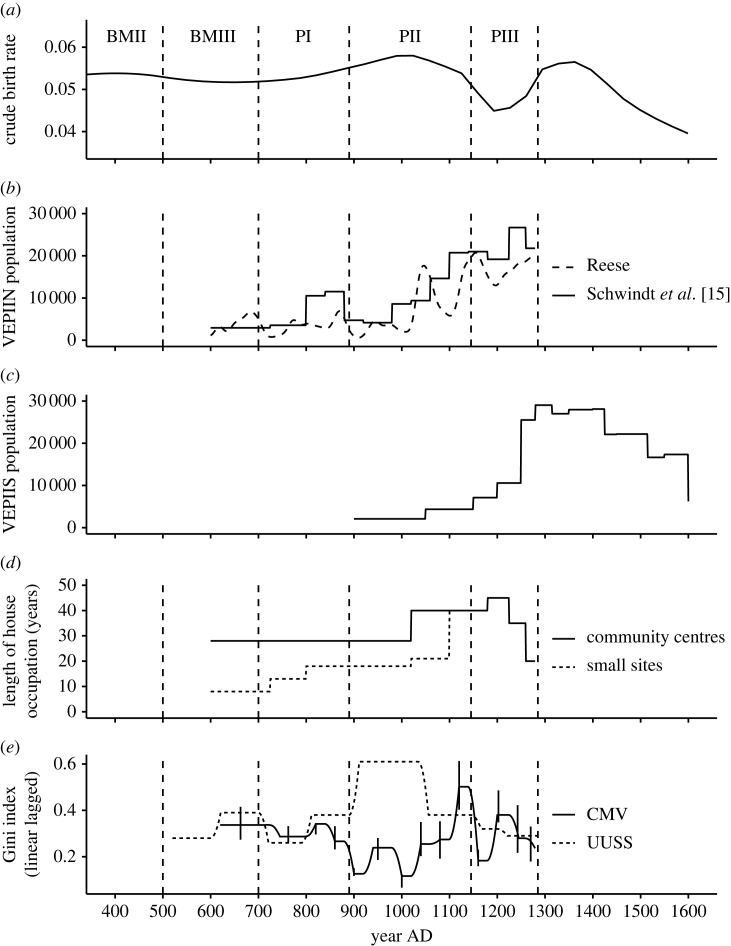
Demographic reconstructions and measures of wealth inequality. (*a*) Crude birth rates estimated from juvenility index, following [38]. Recomputed for the period shown using data from [33] and a loess smoothing with alpha (span) = 0.5. Spatial scope: US Southwest. (*b*) Two estimates of population size. Spatial scope: VEPIIN area. Reese [39]. (*c*) Estimate of population size [16]. Spatial scope: VEPIIS area. (*d*) Estimates for house longevity in small hamlets and villages [14]. Spatial scope: VEPI area. (*e*) Estimates of wealth inequality. Spatial scope: the central Mesa Verde (CMV), Chuskas, Chaco and the Middle San Juan regions [27]. BMII, Basketmaker II; PI, Pueblo I (etc.).

Southwest-wide trends in crude birth rate and life expectancy at age 15 were estimated by Kohler & Reese [33] from a dataset comprising all the well dated and well studied sets of human remains that they could locate in the published and grey literature. The crude birth rate increased slowly from approximately 1100 BC to approximately AD 500, remaining high with some variability to approximately AD 1100. It reached a nadir at approximately AD 1200, and made a final increase to AD 1300, which was followed by a very marked decline (figure 1*a*). From about AD 100 to AD 1400 the crude birth rate appears likely to have exceeded that of the world's fastest-growing country—Niger—in 2013 [33]. Though this conclusion must be treated with caution given the potential biases in the bioarchaeological record, it underscores the remarkable growth achieved by these populations. Life expectancy at age 15 increased very slowly from approximately 800 BC to approximately AD 700, increasing rapidly after that until approximately AD 1000, declining to a nadir at approximately AD 1150, with some increase until approximately AD 1350, followed by another decline [33]. Overall it is reasonable to believe that population growth rates throughout the Southwest were highest between about AD 500 and AD 1000/1100.

The on-the-ground effects of these region-wide rates can be seen in two well-studied subregions, VEPIIN (figure 1*b*) and VEPIIS (figure 1*c*), where we can derive population estimates from estimates of structure counts and of survey coverage. The comparison of these two trajectories reveals the famous depopulation of the northern Southwest (including the VEPIIN area), with migration to the VEPIIS area, and elsewhere, in the thirteenth century AD [16,40]. If we consider them to be representative of the larger Southwest they seem to indicate a population peaking in the AD 1200–1300 range.

### (b) Productivity on this landscape was variable through time and across space, but some field locations were quite dependable

**Figure 2.**
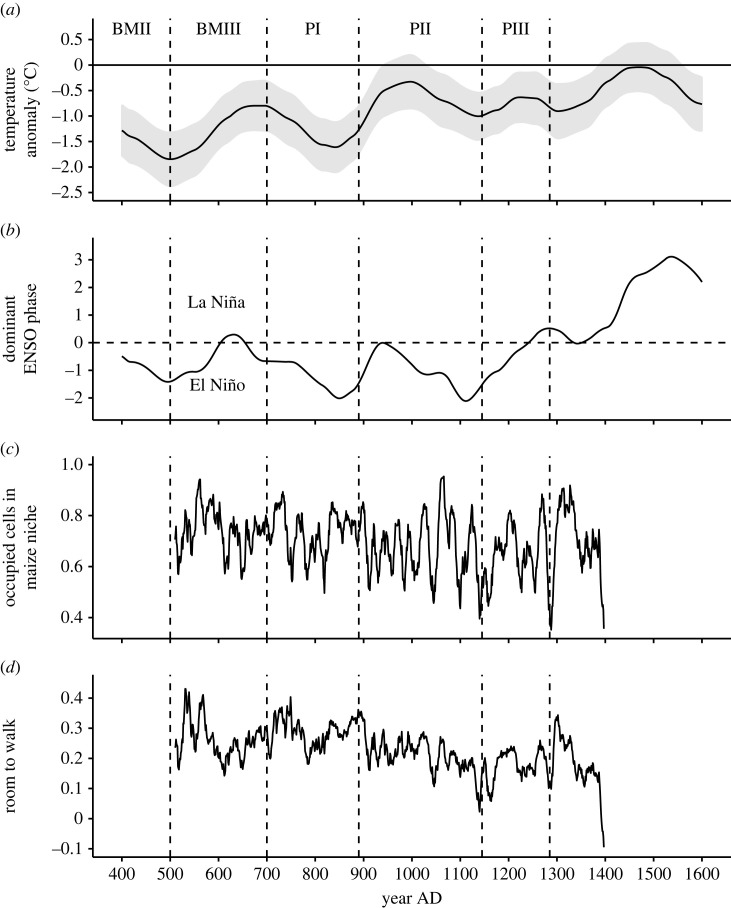
Measures of climate relevant to maize productivity, and estimates of declining freedom to relocate through time. (*a*) Low-frequency estimate of summer temperature derived from pollen [28], relative to modern conditions (preliminary, based on research currently under review). Spatial scope: US Southwest. (*b*) Inferred low-frequency ENSO phase dominance derived from pollen [28] (preliminary, based on research currently under review). Spatial scope: US Southwest. (*c*) Proportion of demonstrably occupied cells (having tree-ring dates indicating occupation in the present or any of the previous 3 years) in the maize farming niche [19]. Spatial scope: UUSS as defined in [19]. (*d*) *Room to walk* as defined in text. Spatial scope: UUSS as defined in [19]. Series in C and D smoothed using a one-sided moving average of the preceding 11 years. BMII, Basketmaker II; PI, Pueblo I (etc.).

Maize was central to the diet of many Southwestern populations by the middle of the first millennium BC [41]. Dependence on this crop increased to the point where, by the end of the occupation of the northern Southwest, R. G. Matson was willing to term it ‘unhealthy’ [42]. Maize productivity was critical to population size and rates of growth. In most parts of the Southwest, soil moisture limits maize production, so maize production will generally be favoured by low-frequency trends towards cooler temperatures (figure 2*a*), which limit evapotranspiration, and El Niño activity (figure 2*b*). In the instrumented record, El Niño years in the Southwest bring above-average winter precipitation [43,44]. As figure 2*a,b* shows, these low-frequency trends were both quite favourable for maize production for nearly all the period covered by these graphs, though the ENSO phase in particular began to turn unfavourable in the mid-1200s.

To characterize potential maize production at higher spatial and temporal resolution, the VEP developed two different estimators. First, for just the VEPI area (a subset of the VEPIIN window), Kohler [17] estimated absolute productivity using methods modified from those developed by Carla Van West [45] and Barney Burns [46].

Another method for characterizing maize productivity was developed by Bocinsky *et al.* [18,19]. This method identifies those locations (cells) on the landscape of the entire Southwest where maize could likely have been grown without management of surface water, and how that changed annually. There are 691 200 such cells within the US Southwest as we defined it, with cells of 30 arcsec (or a little under 1 km^2^) in size. In this method, tree-rings sensitive to temperature are used to characterize the Fahrenheit growing degree days (FGDD) available each year in each cell; other tree-rings are used to estimate the annual water-year (previous October to current September) precipitation for each cell. Cells exceeding 30 cm of precipitation and 1800 FGDD are considered to be in the maize direct-precipitation farming niche.

For the UUSS, figure 2*c* shows the smoothed variability over time in the proportion of cells in the MFN that were currently occupied (as demonstrated by tree-ring dates). This illustrates the temporal variability experienced by these farmers. This proportion is affected both by the changing size of the maize farming niche through time, and by the number of farmers attempting to locate within that niche. The spatial variability in placement of the maize farming niche is illustrated in fig. 1 in [19], which shows the percentage of years each cell in the UUSS is in the maize farming niche between AD 500 and AD 1400; darker green represents more years in the niche. This spatial distribution of productivity suggests that resource endowments for households could have varied significantly. At the same time, there are obviously some locations that were consistently better than others. If these better locations could have been monopolized by certain households or lineages, they might have been able to increase their wealth relative to other households or lineages. Figure 1*d*, however, shows that typical house use-life was sub-generational until about 1020 (for hamlets) though never less than about 28 years in villages (see [14] for details). Such frequent movement likely reduced intergenerational transmission of wealth, especially inheritance of superior fields.

### (c) Room to walk

In the UUSS, where we can draw on the tree-ring record for dating, the locations people chose to build their houses were much more likely to be within the MFN than were randomly chosen locations of the same date in the US Southwest. Kyle Bocinsky and colleagues graph through time the proportion of cells with tree-ring-dated houses in the maize farming niche in the current year or any of the previous 3 years [19]. We reproduce this line for a shorter period, using a different smoothing convention, in figure 2*c*. This line is (almost) always higher than the proportions of all cells in the US Southwest currently in the MFN. In figure 2*d* we plot through time the difference between these two proportions and define it as *room to walk*:

$$\begin{array}{rl} room\;to\;walk & =\frac{n_{\mathrm{currently}\;\mathrm{occupied}\;\mathrm{cells}\;\mathrm{in}\;\mathrm{MFN}\;\mathrm{according}\;\mathrm{to}\;\mathrm{tree}\;\mathrm{rings}}}{n_{\mathrm{currently}\;\mathrm{occupied}\;\mathrm{cells}}}\\ & \;-\frac{n_{\mathrm{cells}\;\mathrm{in}\;\mathrm{the}\;\mathrm{MFN}}}{n_{\mathrm{cells}\;\mathrm{in}\;\mathrm{the}\;\mathrm{US}\;\mathrm{Southwest}}}. \end{array}$$

The first proportion will be high when most occupied cells are in the maize farming niche, as we expect under low populations. This term has a relatively high mean and variance, and is affected primarily by demography and climate. The second term will be high when climates are propitious for maize; it tends to have a lower mean and variance; it is sensitive to climate exclusively. *Room to walk* is simply the difference between the white and black lines in fig. 2*c* of [19]. The purpose for subtracting the second term from the first is to remove most of the climatic variability from *room to walk* so that it is primarily affected by demographic variability. (We consider this metric in more detail in [48]. Note that the tree-ring-based measures of maize niche size used here may not completely reflect the low-frequency processes portrayed in figure 1*a,b*). Decreasing values suggest mounting constraints on ability to find dry-farmable land that is currently unoccupied. As *room to walk* decreases, duration of house occupation (figure 1*d*) increases; note also the regular decline in *room to walk* throughout the PII period especially. We suspect that declining *room to walk* is the primary driver of increasing duration of house use, though other factors such as increasing importance of the web of social relationships to household survival, or political constraints while Chaco was dominant, could have been important as well.

**Figure 3.**
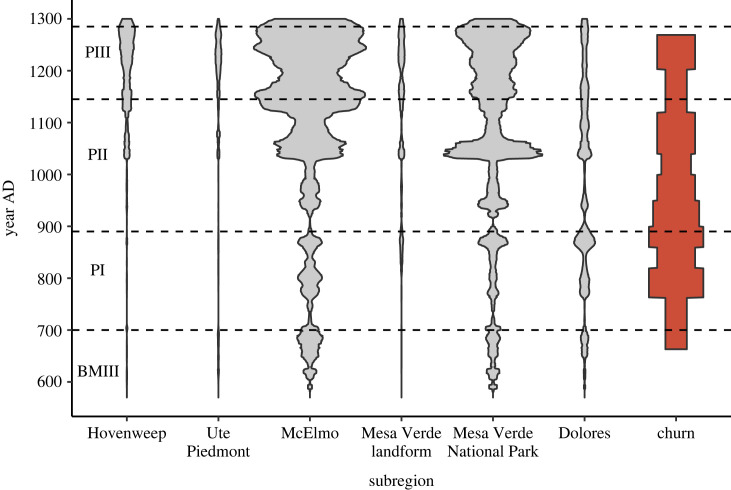
Grey-shaded columns: population size in the VEPIIN area by subregions as defined in [15]. Data from [39]. Red-shaded column: *churn*, a measure of degree of mobility at the subregional scale, using data from [15].

In figure 3 we plot another measure, labelled *churn*, that may reflect similar packing constraints on mobility. Churn is a measure of degree of mobility at the scale of the six VEPIIN subregions as defined by Schwindt *et al.* [15], which average about 680 km^2^ in size—similar in scale to the ‘landscapes’ considered by landscape ecology [48]. In table 5 of [15] we tabulated the population growth rates (per cent per year) from one period midpoint to the next, for each of the 14 VEP periods and each of the VEPIIN subregions. *Churn* counts how many of these rates equal or exceed |0.7| in each period, and is thus bounded between 0 and 6 in any period. George Cowgill [49] argued that population growth, or loss, of this size or larger likely indicates immigration or emigration.

*Churn* cannot be accurately tabulated prior to about AD 725 since house-use lives were much shorter than the length of the first period in the VEP chronology. After about 900, however, *churn* tends to decrease through time until a final increase reflecting the high degree of relocation during the depopulation. Inspection of *churn* beside the violin plots of population size in each subregion through time (figure 3) suggests that most *churn* is caused by movement into or out of the entire VEPIIN area from areas outside it, since there are few times when one of these subregions increases as another decreases. In figure 3, we use measures of population size from [39] whereas the measure of *churn* used the dating of sites and measures of population size developed in [15,50], suggesting that we should use correlations between the two with caution.

### (d) What village (the agent-based model) says about population growth versus resources

We now turn briefly to the VEP modelling domain to illustrate that the increases of population size from AD 600 to AD 900/1000 approximate the maximum growth possible, thus marking a Copial Phase. If one were to take the PTW model and make the minimal modifications necessary to fit it to the CMV region between AD 600 and AD 1300, the result would be something very similar to V2.8 of the ‘Village’ simulation, with the chief difference being that Village has been recast as an agent-based instead of a systems model. In the V2.8 version of Village, as in the PTW model, agents (households) are mostly unable to intensify their subsistence production, or to progress technologically, though admittedly even these simple V2.8 agents can tinker with their resource mix and their exchange practices to make the best of their current local circumstances, in contrast to the populations in the PTW model. The fact that households can also exchange with other households even in this simplest version of Village is already a departure from the insular vision of the members of the population in the PTW model.

Despite these minor intensifications available to them, the agents in V2.8 very clearly run up against a carrying capacity threshold around AD 1000 that is similar to the threshold that the PTW populations reach at the end of their Copial Phase [51]. Interestingly, the population size at which this ceiling is met varies more according to the parameters governing the run than does the date at which the ceiling is reached.

Once the ceiling is reached, a few V2.8 populations (that is, the simulated households in a particular run) continue a slower increase in size, taking advantage of the minor intensifications mentioned above; in most runs household numbers oscillate around sizes reached at *ca* AD 1000, as dictated by the productive conditions they encounter. These in turn are dictated by climate signals proxied by various tree-ring records.

An interesting contrast can be made between the V2.8 Village populations with their very simple agents, and those explored by Crabtree and colleagues [24] using an elaborated version of the same model. The latter agents are capable of much more complicated behaviours, which include forming groups with territories and playing an evolutionary public goods game within these groups; fighting or negotiating with other groups over superior agricultural land, and perhaps forming larger groups as a result; and raising turkeys when hunting wild animals becomes costly owing to depression of game populations. Kohler *et al.* [53] discuss several aspects of this more sophisticated model in detail.

The effects of these changes on global population sizes generated by running the simulation are remarkable [24,53]. In the first place, these more complicated behaviours allow the same landscapes to support more people (agents) as households form groups that continually re-arrange themselves with respect to the changing landscape and the changing locations and natures of other groups. These groups may also create value through their cooperation, depending on their success in the public goods game. Consequently the sharp plateaus in population size seen in the V2.8 simulations are much less visible, and under some parameter settings populations continue to increase almost to the end of the simulation.

An immediate conclusion from this comparison is that the sharp end of the Copial Phase in the PTW model is due to the complete lack, in that model, of human inventiveness, tendency to form internally cooperative groups, and inter-group competition (and its attendant mortality). Another factor that contributes to the sharp threshold at the end of the Copial Phase in the PTW model is that everyone in that model is exposed to exactly the same conditions, whereas in all variants of Village, households are continually reacting to their highly variable local conditions, thus expressing different behaviours. Admittedly even the more complicated agents portrayed in [24,53], however, would eventually reach the end of their available behavioural modifications. It seems likely that this never happens for modern humans [53].

It *is* possible, however, that real populations can grow faster, or undergo climate change that affects their carrying capacity more rapidly, than their inventiveness can successfully react to. The data streams in figures 1 and 2 suggest that this could have been happening to Pueblo populations. Modelling results describe how social complexity allows room for further population growth even after the onset of the Malthusian Transition Interval.

### (e) Nitrogen cycling

Peter Richerson and Robert Boyd suggest that close attention to the way nitrogen is used and cycled in a socioecology can reveal how close a population is to its carrying capacity. ‘If we were to pick a single element that most closely represents the total carrying capacity of the Earth, for life as a whole and for humans in particular, it would be nitrogen’ [2, p. 89].

At least two indices suggest that by the AD 1000s Pueblo populations in the CMV were bumping up against nitrogen limits. First, by that time the most attractive source of hunted protein, deer, was becoming scarce on the landscape. This happens both in the model world of Village [54] and in the real world as analysed by zooarchaeologists [55]. In those versions of Village that allow it, households begin to raise turkeys for meat around that time to help make up for shortfalls in protein [22]. In both model and history, this promotes further reliance on maize, since this is an important source of turkey nutrition [56]. This constitutes a trap in periods when climate caused maize production to decline but human and turkey populations remained high [57].

Second, comparisons between locations of households in the V2.8 version of Village and real household locations in the VEPI area indicate a radical change in the locational practices of households in the early-to-mid-1000s. After the mid-1000s, agents living in a model world where soils degrade very slowly produce a markedly better fit to the locations of real households than do agents living in a world where soils degrade rapidly [21]. Following the mid-900s, agents operating with the constraint that if they move it must be to a place where hunting is likely to be fruitful, produce a better fit than do agents who are not required to prioritize access to game in moving [21]. In fact this often suppresses movement, better reproducing the very stable site locations seen in the last half of the occupation of the VEPI area.

Site stability certainly also had implications for longevity of field use. Larry Benson sampled and analysed soils from several regions in the Southwest densely occupied by prehispanic maize farmers [58]. His field-life calculations for Mesa Verde National Park, taking into account organic-nitrogen values and a range of potential N-mineralization rates, suggest that the most favoured locations could produce 10 bushels of maize per year for only about 18 years under the most favourable conditions (though the best locations in Morefield Canyon within the Park—by far the most potentially productive of any he sampled—could possibly last three times that long). He speculates that stands of bitterbrush (*Purshia tridentata*), a nitrogen-fixing plant that is fairly common in the VEPIIN area, could have slowed depletion of the organic N content of some soils, assuming they were located nearby, upslope, and not eliminated during field clearance. Such calculations underscore the critical importance of nitrogen and the probability that it became a limiting factor in farmers' ability to sustain the long-lived fields that became common in the AD 1000s and beyond.

### (f) Wealth inequality

Following the lead of a rapidly increasing strand of research pioneered by [25,59] we quantify wealth inequality here through Gini coefficients calculated on household-size distributions. ‘Wealth’ is considered to be multidimensional, reflecting differences in embodied, relational and material endowments as defined by Borgerhoff Mulder and colleagues [60]. Figure 1*e* draws on data from [27] to characterize the degree of wealth inequality through time in the CMV, and in the larger northern Southwest. The CMV data have been periodized using the VEP periods [50] and thus are more temporally precise than the data for the larger Southwest, which are periodized by the Explore/Exploit subdivisions of the Pecos stage scheme developed in [19].

The CMV area and the larger northern Southwest exhibit comparable levels of wealth inequality (similar to that calculated for the Hopi pueblo of Oraibi *ca* AD 1900) until about AD 900, when Gini coefficients, driven by the appearance of Chacoan Great Houses, increased markedly for the northern Southwest as a whole while declining in the CMV. Ginis also increased in the CMV in the mid-1000s, as the Chacoan system entered the area, even briefly surpassing those of the larger northern Southwest in the early 1100s. Wealth inequality returned to low levels in the early 1200s in the CMV, but increased to levels beyond that of the larger northern Southwest by mid-century before both declined during the depopulation. For the VEPIIN area, Kohler and colleagues [61] show a statistically significant tendency for periods with high wealth inequality to be followed by periods of high violence, and conversely periods of high violence are followed by periods of high wealth inequality. Although space prevents us from pursuing these relationships here, they suggest that marked wealth inequality then, as now, was socially contentious.

## 3. Wealth inequality, constraints on mobility, and approach to carrying capacity

To summarize, three of our indices mark constraints on household mobility. Duration of house use (figure 1*d*) increases as packing constraints increase, whereas *room to walk* (figure 2*d*) and degree of *churn* (figure 3 right-hand column) tend to decrease as constraints increase. These operate alongside several indications that these populations were approaching carrying capacity. These include the leveling of growth after AD 1000 in the V2.8 Village simulation; the premium being placed on protein after AD 1000 seen in deer depression and widespread raising of turkeys for meat; the likelihood that nitrogen in fields was becoming increasingly limiting as duration of fields increased; the increased nutrient stress and biological disruption reported by bioarchaeologists for the Pueblo II and III periods; and the increased dominance of maize in diets at that same time. All these changes signal the end of the Pueblo world's Copial Phase around AD 1000.

The rise of the Chaco system in the San Juan Basin in the mid-to-late 800s thus takes place towards the end of this phase. Populations were still growing fast. Climates overall were favourable to production as low-frequency El Niño conditions continued to dominate the Southwest (figure 1*b*) as they had done for most of the first millennium AD [28]. High-frequency maize-niche calculations show that the proportion of occupied cells within the maize dry-farming niche was near an all-time high in the mid-to-late 800s. Under these circumstances the advantages of a system that provided rapidly growing communities mechanisms enabling them to cooperate internally and externally more efficiently and reliably would be obvious to all. It has long been obvious from its structures and roads that Chaco somehow enabled large-scale social cooperation within its sphere, and recently developed social network analyses based on ceramic assemblage similarities also underscore dense interconnections across the Chacoan world [62]. The plausibility of the general model for polity growth in growing populations constructed by [24], which foregrounds the importance of force or threats of force—coupled with the apparent *absence* of such strife within the Chacoan polity as it was growing, suggests to us that another important function of the Chacoan system was to suppress conflict among constituent villages over agricultural land.

Still, it is clear that people in the CMV did not embrace the Chacoan system immediately, nor did it grow outside the region immediately surrounding Chaco Canyon itself prior to about AD 1030. In fact, the steep decline in CMV Ginis just as Ginis across the northern Southwest increased suggests that the Chaco model of wealth inequality (and the social/ceremonial system to which that was presumably coupled) was actively resisted. It has even been suggested that some populations in the northern Southwest expanded into the Fremont area around AD 1000 precisely to escape Chacoan domination [63]. Kohler and colleagues [64] have interpreted an anomalous blip in violence in the CMV in the late 900s or early 1000s as resistance to initial Chacoan attempts to expand. Male-biased sex ratios in the CMV in the 1200s coupled with female-biased ratios in Chaco and the Aztec (Middle San Juan) areas in the 1000s and 1200s have been used to suggest that Chaco and its follow-on Aztec raided areas for women that were not yet incorporated in their system [65].

Chaco's post-1030 expansion happened just as the constraints of the Malthusian Transition Interval were beginning to be felt. We suspect that this is not just a coincidence, but that the growing constraints on movement assisted the growing polity in incorporating outlying areas. Perhaps few populations were willing to take the dramatic step of long-distance migration, and opportunities for more local relocation were increasingly limited.

The high levels of wealth inequality witnessed in the 1000s and 1100s did not survive the twin challenges of the mid-1100s high-frequency drought (figure 2*c*) and the continual low-frequency weakening of El Niño conditions and their eventual replacement by droughty La Niña-like conditions in the 1200s (figure 2*b*). Chaco had enough characteristics of a tributary state [66] as to be unable to survive a lack of taxes or tribute flowing to the centre from the periphery [24].

## 4. Postscript

The big picture of the Puebloan Southwest drawn here depicts a series of societies growing rapidly through a Copial Phase from prior to AD 600 to about AD 1000. Innovation in technology appears to have been slower than innovation in the sociopolitical and ceremonial spheres, though the invention of a regional polity towards the end of the Copial Phase provided the organizational tools for growth to continue into the Malthusian Transition Interval even as density-dependent limits began to be felt. History and its contingencies (the Tyche of our title) treated this experiment in complexity harshly, as the maize production on which Chaco depended first faltered in the face of high-frequency drought, and later and more definitively fell victim to the departure of the El Niño-like conditions that had dominated Southwestern climates for a millennium.

Reflecting on what this record suggests about the requirements for inequality more broadly, it seems that patchy resources resulting in different household opportunities, and increasing constraints on mobility are key ingredients. The latter factor encompasses two slightly different potentialities. On one hand it signals difficulty of escaping nascent systems of inequality, and on the other hand increased permanence of settlement makes transmission of superior land within particular lineages more likely. The systems of balanced reciprocal exchange that likely dominated these societies (at least outside any hierarchically imposed system) would have done little to level these societies. However, Kohler & Higgins [25] argued that ceremonial systems may have been effective leveling devices in some Pueblo I societies, and if so, overturning those systems must have been important to the growth of the Chacoan polity. All in all, our results support findings from recent cross-cultural research that social stratification increases significantly as environmental heterogeneity and environmental circumscription increase [67]—though the circumscription in the societies examined here was both demographic and environmental in nature.

As advice for ancient entrepreneurs, in a Neolithic society the best time to found a village-spanning polity is when growth is still very rapid and has not quite become throttled by density-dependent constraints. At that point a polity will look attractive for its ability to move production around to buffer spatial and temporal shortfalls and to suppress wasteful competitive violence among villages within its span of control. Prospective participants may well be ready to give up some autonomy given dwindling outside options, and their labour will be highly attractive to potential patrons as such societies are in the transition from labour-limited to land-limited [67,68]. Further growth will be assisted by the growing difficulty of escaping the system as populations have already expanded into the most productive areas. At some point polities are free to shift from being vehicles of cooperation to embrace a more coercive expansionism. For Chaco this happened around AD 1030 [66]. Given continued generous production Chaco might well have become a fully fledged state. That was, however, not to be.

In this article we have tried to demonstrate that structural conditions in the northern Southwest favoured the rise of a system such as Chaco offered. Almost needless to say, these were not in themselves causal, nor would agency in absence of structural support have been effective [69]; Chaco required both to emerge and succeed for some three centuries. Strong agency was provided, in part at least, by the elite and powerful matriline entombed in the crypt of Pueblo Bonito [70], evidently combining political and spiritual leadership in persuasive combination—for as long as the structural conditions permitted.

## Data Availability

Materials available from the Zenodo repository: https://doi.org/10.5281/zenodo.7562773 [71].
